# A new role of ^11^C‐Choline PET in localizing the epileptogenic foci in insular cortex in the patients

**DOI:** 10.1111/cns.13215

**Published:** 2019-09-11

**Authors:** Yu‐Feng Chen, Ran Wei, Guan‐Qian Yuan, Dan‐Dan Gao, Qing Jin, Xiao‐Yu Cui, Guo‐Xu Zhang, Jia Guo

**Affiliations:** ^1^ Department of Nuclear Medicine General Hospital of Northern Theater Command Shenyang China; ^2^ School of Sino‐Dutch Biomedical and Information Engineering Northeastern University Shenyang China; ^3^ Department of Neurosurgery General Hospital of Northern Theater Command Shenyang China; ^4^ Department of Pathology General Hospital of Northern Theater Command Shenyang China

Epilepsy has been acknowledged as a most common severe neurological disorder, which affects more than 50 million people worldwide.^1^ The pathogenesis of epilepsy is complicated, ranging from single genetic point mutations to metabolic dysfunction, as well as developmental, neoplastic or acquired brain lesions. Despite appropriate therapy with antiepileptic drugs, up to 30% of patients continue to suffer from frequent seizures.^2^ For these patients, epilepsy surgery must be seriously considered since multiple studies have shown that cure of seizures may be achieved by removing the epileptogenic zone. At present, the most epilepsy surgeries are performed in both temporal (50%‐75%) and frontal lobes (25%). However, these operations cannot completely control the induction of seizures, because insular epilepsy is misdiagnosed with temporal, frontal, or even parietal lobe epilepsy that is unfortunately operated in the wrong area,^2^ and the fact that a more complex epileptogenic network includes not only the temporal lobe but also the neighboring insula cannot be recognized comprehensively.

The insular cortex, the fifth and smallest lobe of the brain, is a complex structure enclosed in the depth of the Sylvian fissure covered by the frontal, parietal, and temporal opercula. Due to the difficult access to insular cortex and the high density of blood vessels within the sylvian fissure, the method of electrode implantation has limitations. Moreover, scalp EEG is often misleading, as insular seizures could mimic seizures originating from frontal, temporal, and parietal areas. More recently, in clinical practices, the MRI and positron emission tomography (PET) as noninvasive diagnostic technologies have been widely used to localize the epileptogenic zone for years. However, MRI‐negative cases still account for up to 25% of all patients exposed to presurgical evaluation.^3^ In addition, the major drawback in clinical PET imaging is lack of specificity: In epilepsy, ^18^F‐FDG PET shows both the cause and consequence of seizure activity in the focus and projection area of the seizure onset. This can make treatment decisions for surgery difficult. Moreover, due to the transient of the ictal state, the way to effectively and timely gather the information on the epileptogenic zone by the technology of PET is inconvenient for the clinicians. So, the development of new specific PET radiotracers to identify focal abnormalities during the interictal state and inform surgical treatment has been a long‐term aspiration in epilepsy surgery. Choline, as an endogenous substrate, plays the main physiological role in participating into the processes of biochemistry, such as structural integrity and signaling roles for cell membranes.^4^ In addition, choline has been widely used as a radiotracer in the diagnosis of primary prostate cancer and low‐grade astroglioma, which suggests that the proliferation of tumor cells, astroglioma in particular, can contribute to the hypermetabolic level of choline. Therefore, based on the theory that neuronal loss or dysplasia always companies with astrogliosis in the epileptogenic zone, and choline can be conducive to synthesis of cell membrane during the process of astrogliosis,^5^ we selected ^11^C‐Choline as the new radiotracer for diagnosis of insular epilepsy in the interictal state and further explored its clinical values for providing the new insight into accurately localizing the epileptogenic zone in the present study.

We enrolled nine patients who were diagnosed as epilepsy and further suspected of insular epilepsy based on both scalp video‐EEG monitoring data and the typical clinical semiology (Table 1). Those met these following criteria: 1. At least one scalp video‐EEG spike sources were observed (anterior and/or posterior operculoinsular cluster, and/or diffuse perisylvian scatter); 2. All of those had suffered from at least one of the following symptoms: (a) the consciousness of patient was not lost completely at the beginning of the attack and could response to the surrounding environment; (b) somatosensory symptoms, such as the sensation of tingling, warmth, tension, or electrical current, could involve either large cutaneous territories or be restricted to an limited area; (c) visceral movement and visceral sensory symptoms were shown, such as nausea, vomiting, the tightness of the throat, and a feeling of pressure behind the sternum and the abdomen, in accompany with a large amount of saliva over‐secreted and the abnormal heart rhythm; (d) gustatory or auditory hallucinations and postictal aphasia were shown.

**Table 1:** Information of nine patients with insular epilepsy

No.	Age(y),Sex	MR	^11^C‐Cho PET/CT	SEEG (No. of electrodes)		Operation	Pathology of insula	Seizure Outcome (Engel)
				Total	Insula			
1	21,F	Insula(Lt)＆OPL(Lt) ＆TL(Lt)	Insula(Lt)＆OPL(Lt)	12	4	Insula(Lt)＆OPL(Lt)	MCD	Engel I
2	25,M	‐	Insula(Rt)	13	3	Insula(Rt)	MCD	Engel I
3	10,F	Insula(Rt)＆TL(Rt)	TL(Rt)＆Insula(Rt)	8	2	Insula(Rt)＆TO(Rt)	MCD	Engel I
4	18,M	hippocamp(Lt)	Hippocamp(Lt)＆Insula(Lt)	12	4	Insula(Lt)	MCD	Engel III
5	30,F	FL(Lt)＆TL(Lt)	Insula(Lt)＆TL(Lt)	13	2	Insula(Lt)＆TL(Lt)	MCD	Engel I
6	22,F	Insula with OL(Lt)	Insula(Lt)	8	2	OL in the insula	glioma	Engel I
7	39,M	FL(Rt)	Insula(Rt)＆FO(Rt)	14	2	Insula(Rt)＆FO (Rt)	MCD	Engel I
8	55,F	‐	Insula(Rt)	15	3	Insula(Rt)＆TO(Rt)	MCD	Engel II
9	23,F	TL(Lt)	TL(Lt)＆Insula(Lt)	17	5	TO(Lt)＆Insula(Lt)	MCD	Engel I

Abbreviations: FL, Frontal Lobe; FO, Frontal Operculum; Lt, Left; MCD, Malformation of Cortical Development; OC, Orbital Cortex; OL, Occupying Lesion; OPL, occipital lobe; Rt, Right; TL, Temporal Lobe; TO, Temporal Operculum.

According to the protocols described in the previous work,^6^ the high‐resolution MRI brain scans were performed in the nine patients with 3.0 T MR scanner (MR 750 Discovery, General Electric Company), including axial three‐dimensional (3D) T1‐weighted sequences (Figure S1A), axial and coronal T2‐weighted sequences (Figure S1B), and axial and coronal fluid‐attenuated inversion recovery (FLAIR, Figure S1C). The results showed that there were a T1 hypointense and T2/FLAIR hyperintense lesion in the left‐side insular cortex in one patient, indicating that tumor might exist, and there was the normal surface area of bilateral insular cortex or the abnormal surface area of unilateral insular cortex without identified lesions in the remaining eight patients.

It's well known that the anatomical structural changes of tissues and organs are triggered by the abnormal levels of metabolism which can't be exhibited by the method of MRI brain scans.^7^ In order to testify the results from scalp EEG and exclude the false‐negative results of MRI, 3D dynamic ^11^C‐Choline PET/CT brain scans were performed in those recruited. Each patient was restrained on the examination bed of PET/CT scanner (Discovery VCT, General Electric Company) was administered intravenously at the dose of 259 ~ 555 MBq (3.7 ~ 5.55 MBp/kg) ^11^C‐Choline. Brain PET/CT scanning started as soon as injection was finished. CT images with 3.7 mm spatial resolution were acquired with 64‐slice CT detectors, and PET ones were captured with lutetium oxyorthosilicate crystal detectors 5 minutes per frame for 60 minutes. The postprocessing of captured image was conducted with Xeleris Functional Imaging Workstation (General Electric Company) to accomplish iterative reconstruction in the axial, coronal, and sagittal planes, in accompany with CT being used for attenuation correction. The results from ^11^C‐Choline PET/CT images showed that there was hypermetabolism of choline in the left‐side insular cortex in the patient suspected of tumor lesions diagnosed by MRI in the first 20 minutes, which could confirm that the patient was afflicted with low‐grade gliomas. A series of literatures have reported that ^11^C‐Choline PET has been successfully applied as an accurate diagnostic tool for identification and detection of low‐grade gliomas and meningiomas. Moreover, the low‐grade brain tumors, such as ganglioglioma, dysembryoplastic neuroepithelial tumor, angiocentric glioma, and isomorphic astrocytoma, associates with early epilepsy onset.^3^ However, in the remaining eight patients, large quantity of choline accumulated slowly in the unilateral insular cortices during the period of 60 minutes, and there was significant difference in the time‐radioactivity curves of choline between bilateral insular cortices from about 40 minutes after injections (Figure S1D). It might attributed to the fact that although the ^11^C‐Choline could enter freely the zone of brain when the blood‐brain barrier was broken in the epileptic patients, it accumulated in the epileptogenic foci to participate in the pathophysiological processes in a relative long time due to the competition with the endogenous one.

The halftime of ^11^C is approximate 20 minutes. In the present work, it took approximate 3 halftimes to capture PET/CT images of the ^11^C‐Choline, so that the captured images showed slightly low resolution due to radionuclide decay. In fact, as it was difficult to find the region of interest (ROI) of insular cortex in the PET image by visual inspection, we were planning to develop the applicable software to conduct the computer‐aided diagnosis of the epileptogenic foci marked by ^11^C‐Choline. The images were segmented based on the mathematical modeling with respect to the same pixel of different 12 points in time. Moreover, image preprocessing was necessary to improve the accuracy of the image segmentation. Firstly, the 2‐D median filtering was used to reduce the noise of the images. A patient's dataset consisted of 564 images which were divided into 12 groups. Each group had 47 different layer images, and the dimension of each image was 128 × 128. After choosing the images of the same layer among 12 groups, we got 12 images of the same layer, and the only difference among them was time. Because the concentration of contrast agent had a linear and positive relationship with the gray level of each pixel, the gray level value could be used to analyze the changing of the concentration of the contrast agent. Secondly, we collected the same pixel of the 12 images so that we got 12 gray level values as a sequence. As the dimension of image was 128 × 128, we could get 128 × 128 sequences for a certain layer and there were 12 points in each sequence. There were many pure mathematic models which could fit the sequence. Among them, quintic polynomial $C\left(t\right)=\sum_{i=1}^{5}a_{i}t^{i}$ had been selected to be used. In theory, each sequence produced a quintic polynomial model and the differences between these models were the five parameters of the model, which could establish a curve of the quintic polynomial model. The segmentation of this experiment was accomplished based on the normalized root mean square error （NRMSE）between the fitting curve and the original sequence. The formula to calculate NRMSE was as follows:

$\text{NRMSE}=\sqrt{\frac{\sum_{i=1}^{N}{\left(C\left(t\right)-C_{\text{fitting}}\left(t\right)\right)}^{2}}{N}}$ (*N* is the number of points in a sequence).

Finally, we could get a NRMSE color map demonstrating the regional of interest. The infusion of CT and the color map was done in order to get a more accurate location of ROI (Figure S1E). A light green area on a dark blue background was confirmed in the region of insular cortex in reference to the anatomical map of brain.

In order to further confirm the reliability of ^11^C‐Choline in the diagnosis of epileptogenic foci, ^11^C‐Choline PET/CT‐guided intracranial electrodes were implanted in the regions of lesions and their neighborhoods (Figure S1F). The protocols of electrode implantation had been described in detail in the previous literatures.^8^ Nine patients underwent implantation of a total of 112 electrodes (8‐17 electrodes per patient). Eighty‐nine electrodes (79.5%) were implanted orthogonally through the frontoparietal lobes or temporal operculum. Twenty‐three electrodes (20.5%) were implanted by means of an oblique trajectory either through a frontal or a parietal entry point. When seizure activity started, the ictal spikes were recorded (Figure S1G). The data from stereo‐EEG evaluation showed that the epileptic foci were characterized by a complex epileptogenic network in those 9 patients. More precisely, there were temporoperisylvian patterns (propagation of epileptic discharges from the insula to the suprasylvian operculum) in 6 patients, temporo‐parietooccipital patterns (propagation of epileptic discharges from the insula to temporo‐parietooccipital junction) in 2 patients and temporo‐frontal pattern (propagation of epileptic discharges from the insula to the orbitofrontal cortex) in 1 patient. In addition, none of the patients experienced any intracerebral hemorrhage related to the insular electrodes. These results indicated that the locations of abnormal discharges during the seizure were in consistence with those labeled by ^11^C‐Choline in the PET/CT images.

After the epileptogenic zones were confirmed, brain surgeries were performed, and the resected tissue samples were performed pathological investigation (Figure S1H). All samples were fixed in the 4% paraformaldehyde for 10 hours, and embedded in paraffin at 60°C. 5‐μm‐thick sections of tissue samples were stained with anti‐GDNF and anti‐NeuN antibodies, respectively.^9^ All the sections were observed using a microscope equipped with a digital camera (BX51; Olympus), and five immunostaining images of each section were taken randomly at ×400 magnification in a blinded manner. The immunohistochemical results indicated that NeuN immunoreactivity highlighted the focally thinned cortical ribbon with laminar neuronal cell loss and barely discernible gray/white matter boundaries (Figure S1I). Numerous dysmorphic neurons with enlarged nuclei and aggregates of Nissl substance lacking a regular anatomic orientation were demonstrated. There was numerous reactive gliosis in neocortical and subcortical areas (GDNF‐staining) (Figure S1J). Balloon cells were detected in deep cortical layers as well in the underlying white matter. The balloon cells were characterized by a large cell body with opaque cytoplasm and occasionally multiple nuclei. These data suggested that these samples were actual pathological tissues which could produce the abnormal discharges and testified further ^11^C‐Choline could accurately mark the epileptic foci.

Postsurgery seizure outcomes were scored, according to Engel classification system.^10^ Eighteen‐month follow‐up results from nine registered postoperative outpatients were collected. The results of follow‐up indicated that 7 out of 9 patients were free of disabling seizures or auras within 18 months, who were classified as the favorable outcomes (Engel Class I). 1 out of 9 postsurgery patients had suffered no more than 3 seizures (Engel Class II), and the frequency of seizures decreased about 75% in the remaining 1 patient (Engel Class III). The last two patients were classified as the unfavorable outcomes. These data indicated that the localizations of epileptogenic zones marked by ^11^C‐Choline in the PET/CT images were accurate, which makes the surgeries could achieve satisfactory outcomes.

In conclusion, ^11^C‐Choline possesses the invaluable prospect for localizing the epileptogenic foci in the insular cortex which could be diagnosed negatively by MRI in particular.

## CONFLICT OF INTEREST

These authors declare no conflict of interest.
