# Supplementary material for: A new role of 11C‐Choline PET in localizing the epileptogenic foci in insular cortex in the patients
Source: CNS Neurosci Ther. 2019 Sep 11;26(1):144–7. doi: 10.1111/cns.13215 (PMC6930826; doi:10.1111/cns.13215)
Supplement: Supplementary file 1 [file CNS-26-144-s001.doc]

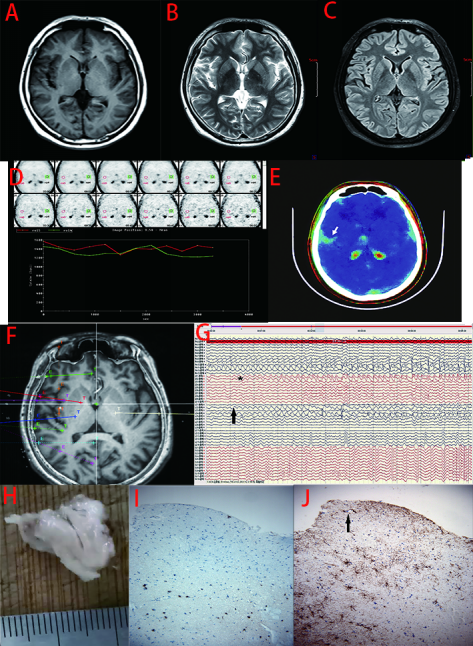


Fig. A 25-year-old male with refractory drug-resistant epilepsy was characterized by nausea, vomiting, the sensation of tingling around mouth, difficult breathing and the abnormal heart rhythm during the seizure. The results from MRI showed no obvious abnormal signals scanned by T1 and T2 weighted MRI sequences and FLAIR sequences (A～C). However, based on the time-radioactivity curves of choline between bilateral insular cortices (D), the fusion of CT and the NRMSE color map showed a ROI region (arrow) in the insular cortex in the PET/CT images (E), indicating that the epileptogenic foci might exist. Then, Choline PET/CT-guide intracranial electrodes were implanted in the regions of lesions and their neighborhoods (F). The stereo EEG showed the abnormal clusters of spikes (arrow, G) earliest in the long gyrus of insula, and the spikes propagated subsequently to the second short gyrus of insula in a very short time (asterisk, G), suggesting that insula was the epileptogenic focus. The immunohistological data from insula sample (H) exhibited that NeuN-immunoreactivity highlighted the focally thinned cortical ribbon with laminar neuronal cell loss and barely discernible grey/white matter boundaries. Numerous dysmorphic neurons were demonstrated (I). There were numerous reactive gliosis in neocortical and subcortical areas (GDNF-staining) (J). Balloon cells were detected in the cortical layers (arrow).
